# The mortality and trends of lung cancer among females in urban and rural China, 2004 to 2018: A cohort study

**DOI:** 10.1097/MD.0000000000046440

**Published:** 2026-05-12

**Authors:** Hong Liu, Yanlong Shi, Lihui Zhao, Gaoyu Cui, Dequan Wang, Xudong Li, Yuewei Chen

**Affiliations:** a Oncology Department, Shandong Armed Police Forces General Hospital, Jinan, China; b South Hospital Regional oncology Dept,The 960th Hospital of the PLA Joint Logistics Support Force, Jinan, China; c Department of Gynaecology and Obstetrics, Shandong Armed Police Forces General Hospital, Jinan, China; d Department of Disease Control and Prevention, Shandong Armed Police Forces General Hospital, Jinan, China; e Office of Epidemiology, Chinese Center for Disease Control and Prevention, Beijing, China.

**Keywords:** female, lung cancer, mortality, trends

## Abstract

Lung cancer is the most common cancer among females and leading cause of cancer-related death among Chinese females. This cohort study aimed to analyze the mortality rates and temporal trends of lung cancer among Chinese females from 2004 to 2018, with a focus on urban-rural disparities. Data were collected from the disease surveillance points system, which monitors major disease imposing a heavy burden on the Chinese population. Annual data were collected across multiple regions and this study extracted lung cancer mortality data. The changing trend of the mortality was described. The mortality of lung cancer among urban and rural female was compared. This study showed the crude mortality rates for females increased from 2004 to the 2018, but age-standardized rates decreased in urban areas and increased in rural areas. In urban areas, the annual percentage change in mortality was −1.2%, and for rural areas, the annual percentage change was 1.2%. Lung cancer mortality increased with age, especially after 50 years of age. This study highlights the need to strengthen lung cancer prevention among females, especially in rural areas. It will be important to target prevention strategies at reducing risk factors for lung cancer among females, especially among those aged under 50 years.

## 1. Introduction

Lung cancer is the leading cause of cancer-related deaths for males and females globally.^[1]^ According to GLOBOCAN, there were approximately 2.1 million new cases of lung cancer and 1.8 million lung cancer deaths worldwide in 2018. Lung cancer is a common cancer and a major cause of death - higher in males than in females in China.^[2]^ From 1991 to 2013 the annual percentage change (APC) in lung cancer mortality was 7.7%, and some studies suggested that this trend would persist for the next 5 years.^[3]^ The highest cancer mortality rates were predicted for lung cancer for both sexes among the European Union.^[4]^ China is one of the most populous countries in the world. And there are great differences in population, economy and environment among different regions. There are great differences in the incidence of lung cancer between female and males. The incidence rate and the mortality of lung cancer is higher in males than in females.^[5]^ Many females suffer from cancer related to passive smoking. There is a lack studies about the females in different regions. Additionally, there remains a knowledge gap for the lung cancer between the city and rural areas, which may lead to different health behaviors and subsequent mortality rates. This study aimed to analyze differences in lung cancer mortality trends among Chinese females in urban and rural settings from 2004 to 2018, using nationally representative data. Understanding these disparities is crucial for informing resource allocation and targeted public health interventions.

## 2. Methods

Ethical approval was obtained from the Ethics Review Committee of the Chinese Center for Disease Control and Prevention. Informed consent was acquired from all participants. Data were extracted from the disease surveillance points system from 2004 to 2018 in China. The disease surveillance points system serves as a key platform for monitoring disease burden, collecting national disease burden data. All provinces in China collected data via census including indicators of incidence, mortality, prevalence. Data were classified and summarized by demographic characteristics such as age, sex and region. We analyzed female lung cancer mortality data, stratifying by urban and rural residence. Crude mortality rates (CMR) were calculated, and age-standardized mortality rates (ASMR) were determined using the 2000 Chinese standard population. Joinpoint regression analysis was conducted to identify significant temporal trends, with APC and average APCs (AAPC) calculated for the 2004 to 2018. Age-specific mortality rates from 0 to 85 years were analyzed. Statistical significance was defined as *P* <.05. All analyses were conducted using R software (v4.2.2).

## 3. Results

**Figure 1.**
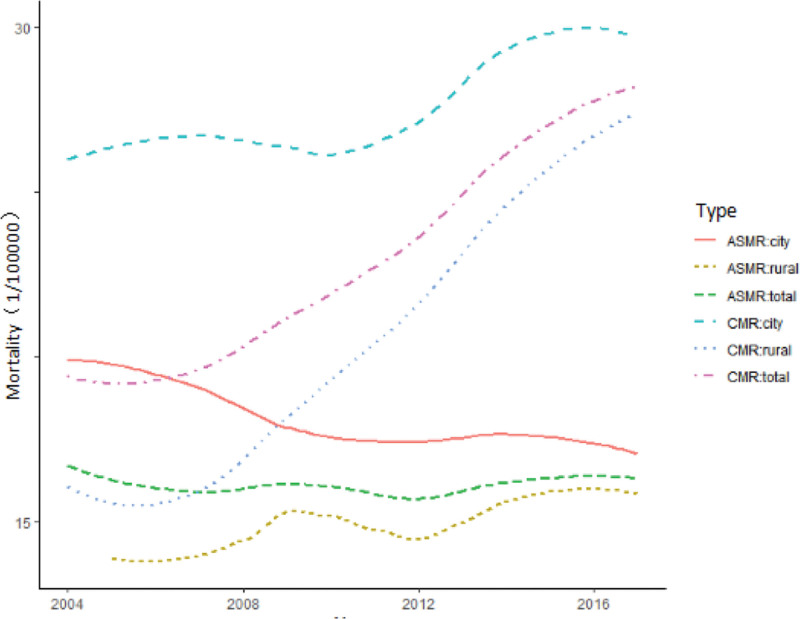
Lung cancer mortality rate among females in China, 2004 to 2018.

The crude mortality rate (CMR) from lung cancer among all females increased from 19.24/100,000 to 28.42/100,000 from 2004 to 2018. Among urban-dwelling females, CMR increased from 25.49/100,000 to 30.13/100,000; among rural-dwelling females, CMR increased from 16.04/100,000 to 27.53/100,000. Notably, ASMR showed divergent patterns: ASMR decreased from 18.94/100,000 to 16.26/100,000 in urban areas, whereas it increased from 14.97/100,000 to 15.45/100,000 in rural areas (Fig. 1).

Joinpoint regression analysis revealed that the overall lung cancer ASMR decreased annually from 2004 to 2006 (AAPC: −3.8%; 95% CI: −12.0% to 6.5%; *P* = .4) and increased from 2006 to 2018 (AAPC: 0.4%; 95% CI: −0.35% to 1.24%; *P* = .3). However, urban and rural populations exhibited significant divergences: urban areas experienced an annual ASMR decrease of 1.2% (*P* < .01), while rural areas showed an annual increase of 1.22% (95% CI: 0.32% to 2.21%; *P* < .01) (Table 1).

**Table 1:** Lung cancer mortality rate (1/1,00,000) for females in China, 2004 to 2018.

Year	Total		Rural		Urban	
	CMR	ASMR	CMR1/10^5^	ASMR	CMR	ASMR
2004	19.24	16.49	16.04	14.28	25.49	18.94
2005	20.43	17.40	16.69	14.97	27.74	21.58
2006	17.47	14.57	13.58	12.06	25.54	18.94
2007	19.80	16.06	15.83	13.71	27.13	19.86
2008	20.26	15.73	17.28	14.61	25.70	17.36
2009	21.74	16.53	18.27	15.11	27.95	18.50
2010	22.07	16.57	19.64	15.97	25.99	17.32
2011	22.03	15.24	19.89	14.10	25.22	17.54
2012	23.61	15.65	21.61	14.51	26.59	17.42
2013	24.86	15.65	22.81	14.85	29.38	17.24
2014	26.69	16.50	25.18	15.81	29.90	17.97
2015	27.69	17.22	26.40	16.74	30.40	18.21
2016	27.61	16.05	26.66	15.62	29.49	17.05
2017	28.05	16.21	27.19	15.84	29.71	17.06
2018	28.42	15.69	27.53	15.45	30.13	16.26
APC(%)						
2004–2006	−3.9 (−14 to 7.4)		1.2 (0.3–2.2)*		−1.2 (−1.8 to −0.6)*	
2006–2018	0.3 (−0.4 to 1.2)					

APC = annual percentage change of the ASMR, ASMR = age-standardized mortality rates, CMR = crude mortality rate.

* : *P* <.05 was considered statistically significant.

**Figure 2.**
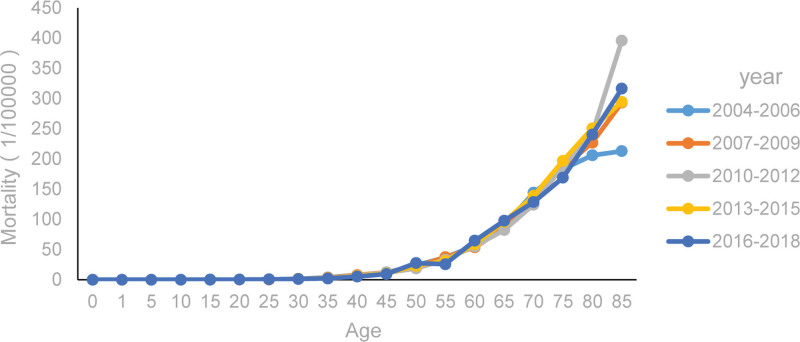
Age-specific-mortality of lung cancer for females 0 to 85 yr old in China.

Lung cancer mortality rates increased with age. After 50 years of age, the mortality rate increased more rapidly, reaching its highest point at 85 years (Fig. 2).

## 4. Discussion

Numerous epidemiological studies on lung cancer have been conducted in China. While males have higher mortality rates than females, female mortality rates are rising faster.^[6]^ Our study analyzed trends and characteristics of lung cancer mortality among females. Between 2004 and 2018, lung cancer trends can be divided into 2 parts - from 2004 to 2006, showing a decreasing trend (AAPC = −3.8), and after 2006, showing an increasing trend (AAPC = 0.4). Between 2004 and 2018, however, APCs of lung cancer mortality diverged significantly between urban and rural females, with statistically significant temporal trends. Mortality decreased 1.2% per year among urban female (*P* <.01) and increased 1.2% per year (*P* <.01) among rural females.

There are several possible explanations for the difference between urban and rural female’s lung cancer trends. Significant geographical inequalities exist in China’s healthcare system. Urban centers typically possess well-resourced hospitals facilitating early diagnosis and treatment. Conversely, rural populations face barriers including lower incomes, limited healthcare access, and reduced awareness, often resulting in later-stage diagnoses and higher case-fatality rates.^[7]^ Another potential explanation is that China launched the New Cooperative Medical Scheme in 2003,^[8–10]^ which improved funding standards and reduced the healthcare financial burden for low-income families. Following several years of development, the New Cooperative Medical Scheme expanded to most rural areas, improving rural residents’ access to diagnostic services. This improved access has increased the likelihood of cancer diagnosis, indirectly raising cancer registration rates and the observed lung cancer mortality rate.

In our age-stratified analysis (0–85 years), we found that females under 50 years had a slight upward trend in mortality, whereas those over 50 years exhibited a far more pronounced increase. The highest mortality rate was observed in females aged 75 to 85 years. The rising mortality rate correlates inversely with human immune system function. Immunosenescence, combined with lung cancer risk factors such as smoking,^[11]^ may contribute to this trend. In China, the smoking prevalence among females increased from 2.3% in 2008 to 2.7% in 2013, with a higher growth rate than that among males.^[12]^ Additionally, e-cigarette use has also increased.^[13]^ These factors, coupled with the significant life pressures faced by females over 50 years of age, may contribute to the observed upward mortality trend. Thus, targeted screening for high-risk groups should be implemented before 50 years of age, health education should be strengthened for those over 50, and active treatment should be provided for the elderly population^[14]^

## 5. Conclusions

In summary, lung cancer mortality increased among rural females, and age-related disparities in lung cancer mortality were observed. Targeted preventive measures – including reducing smoking rates and expanding early screening, detection, and treatment programs – should be implemented to alleviate the burden of lung cancer among Chinese females.

## 6. Strengths and limitations

This study comprehensively analyzed the disease burden of lung cancer among Chinese women, highlighted urban-rural disparities in this burden, and provided a basis for optimizing the allocation of medical resources. However, this study has several limitations. The absence of relevant data in some rural areas may have affected the accuracy of urban - rural disparity analyses in disease burden. Additionally, the failure to explore certain risk factors limits the ability to identify the underlying causes of these disparities. Future studies should incorporate environmental factors and lifestyle changes to better identify potential interventions.

## Author contributions

**Conceptualization**: Lihui Zhao.

**Data curation**: Lihui Zhao.

**Formal analysis**: Dequan Wang.

**Investigation**: Hong Liu, Dequan Wang, Xudong Li.

**Methodology**: Yanlong Shi.

**Software**: Gaoyu Cui.

**Supervision**: Hong Liu, Yuewei Chen.

**Visualization**: Gaoyu Cui.

**Writing – original draft**: Yanlong Shi.

**Writing – review & editing**: Yuewei Chen.
